# Mapping function from FACT-B to EQ-5D-5 L using multiple modelling approaches: data from breast cancer patients in China

**DOI:** 10.1186/s12955-019-1224-8

**Published:** 2019-10-15

**Authors:** Qing Yang, Xue Xin Yu, Wei Zhang, Hui Li

**Affiliations:** 1 Institute of Hospital Management, West China Hospital, Sichuan University, Chengdu, 610041 China; 2 Sichuan Cancer Hospital & Institute, Sichuan Cancer Center, School of Medicine, University of Electronic Science and Technology of China, Chengdu, 610041 China

**Keywords:** Mapping, Health utility, Breast cancer, EQ-5D-5 L, FACT-B, Quality of life

## Abstract

**Background:**

The Functional Assessment of Cancer Therapy-Breast (FACT-B) is the most commonly used scale for assessing quality of life in patients with breast cancer. The lack of preference-based measures limits the cost-utility of breast cancer in China. The goal of this study was to explore whether a mapping function can be established from the FACT-B to the EQ-5D-5 L when the EQ-5D health-utility index is not available.

**Methods:**

A cross-sectional survey of adults with breast cancer was conducted in China. All patients included in the study completed the EQ-5D-5 L and the disease-specific FACT-B questionnaire, and demographic and clinical data were also collected. The Chinese tariff value was used to calculate the EQ-5D-5 L utility scores. Five models were evaluated using three different modelling approaches: the ordinary least squares (OLS) model, the Tobit model and the two-part model (TPM). Total scores, domain scores, squared terms and interaction terms were introduced into models. The goodness of fit, signs of the estimated coefficients, and normality of prediction errors of the model were also assessed. The normality of the prediction error is determined by calculating the root mean squared error (RMSE), the mean absolute deviation (MAD), and the mean absolute error (MAE). Akaike information criteria (AIC) and Bayes information criteria (BIC) were also used to assess models and predictive performances. The OLS model was followed by simple linear equating to avoid regression to the mean.

**Results:**

The performance of the models was improved after the introduction of the squared terms and the interaction terms. The OLS model, including the squared terms and the interaction terms, performed best for mapping the EQ-5D-5 L. The explanatory power of the OLS model was 70.0%. The AIC and BIC of this model were the smallest (AIC = -705.106, BIC = -643.601). The RMSE, MAD and MAE of the OLS model, Tobit model and TPM were similar. The MAE values of the 5-fold cross-validation of the multiple models in this study were 0.07155~0.08509; meanwhile, the MAE of the TPM was the smallest, followed by that of the OLS model. The OLS regression proved to be the most accurate for the mean, and linearly equated scores were much closer to observed scores.

**Conclusions:**

This study establishes a mapping algorithm based on the Chinese population to estimate the EQ-5D-5 L index of the FACT-B and confirms that OLS models have higher explanatory power and that TPMs have lower prediction error. Given the accuracy of the mean prediction and the simplicity of the model, we recommend using the OLS model. The algorithm can be used to calculate EQ-5D scores when EQ-5D data are not directly collected in a study.

## Background

Breast cancer has a devastating effect on global population health, accounting for 0.52 million annual deaths [1]. In China, the morbidity rate of breast cancer significantly outweighs that of other cancers, although the survival rate of breast cancer patients has dramatically increased with the development of clinical practice and disease management [2]. As survival rates have improved, health-related quality of life has gained significant attention recently, since the vast majority of survivors suffer from a loss of functions, including arm activity, sexual activity, and sleep quality [3–5]. At the same time, the growing number of survivors and the comprehensive application of advanced medical technology are increasing the burden of the disease on society [6], which calls for an economic re-evaluation, such as a cost-utility analysis.

Extensive generic non-preference-based questionnaires were administered in a prior study to measure health-related quality of life; these questionnaires included the SF-36 [7], the EORTC [8], the QLQ-C30 [9], the IBCSG, the WHO-QOL BREF, and the FACT-B. For breast cancer patients, the chief among these is the FACT-B, which can most accurately measure quality of life [10]. Non-preference-based questionnaires are not appropriate for a cost-utility analysis, since their results cannot derive quality-adjusted life years (QALYs) directly. The QALY is a widely used measure of health improvement that is used to guide health-care resource allocation decisions [11]. Therefore, preference-based measures are highly recommended in health-economic evaluations, such as the EQ-5D and SF-6D, for these measures can directly assess health utility [12, 13].

Although generic preference-based measures, especially the EQ-5D, are highly recommended, they are usually excluded in clinical trials [14]. A recent systematic review of breast cancer health utility values in China found that all three studies of health economics models in China cited measurements of breast cancer patients in the UK or Hong Kong, China [15]. The lack of health utility values limits the development of health economics research. One potential solution is to perform a mapping function, mapping from non-preference-based to preference-based measures. Although a mapping function would lose some information and increase uncertainty, it is currently the only solution for conducting a cost-utility analysis when straightforward health utility data are unavailable [16]. Therefore, it is of great significance to establish a health utility value mapping model for Chinese populations, which can broaden the sources of health utility values and provide important parameters for health economics evaluation.

Currently, there are 3 studies focusing on mapping from FACT-B to EQ-5D [16]. Two prior studies from Singapore performed mapping functions from FACT-B to EQ-5D-5 L among breast cancer patients, and applied the utility-scoring systems, which were derived from data of British and Japanese patients [17, 18]. A study from the UK used the EQ-5D-3 L scale and UK tariffs [19]. However, the study pointed to disparities in utility-scoring systems among nations, owing to different utility weights [20]. Therefore, a utility-scoring system of one nation will not necessarily be applicable to other nations.

A prior study demonstrated that several socio-demographic and clinical factors are associated with the health utility of breast cancer patients [21–23], including age, gender, income, education, and treatment. It is unknown whether these factors could generalize to the overall Chinese population.

The present study will first develop three sophisticated mapping functions from the FACT-B to the EQ-5D-5 L using three modelling algorithms, including the ordinary least squares (OLS) model, the Tobit model, and the two-part model (TPM). Second, this study will compare the predictability of the three models, and the resulting data will be used to select the most appropriate model for this purpose.

## Method

### Participants

The study included 446 breast cancer patients meeting the following criteria:1) diagnosed by pathology or clinical tests; 2) aged 18 or above; and 3) indicated no mental disorders, thus demonstrating full communicative capacity. Patients with severe chronic comorbidities, including cardiovascular and psychiatric disorders, were excluded from this study. All patients came from a tertiary oncology hospital in West China.

Informed consent from all participants was obtained prior to the study. Ethical permission was granted by the Ethics Committee, West China School of Medicine/West China Hospital, Sichuan University (approval number 2017–255).

### Data source

Health-related quality of life data were sourced from two measures, namely, the FACT-B and the EQ-5D-5 L. Demographic data came from field investigation, while clinical data were obtained from electronic medical records. Data were collected in the period from November 2017 to May 2018. Data was collected by the research team. To ensure data qualification, a data collection manual was prepared and the interviewers were trained strictly prior to data collection.

### Independent variables

#### Health-related quality of life from the FACT-B

The FACT-B contains 37 questions along five dimensions: physical wellbeing (PWB), social/family wellbeing (SWB), emotional wellbeing (EWB), functional wellbeing (FWB), and additional concerns about breast cancer (BCS) [24]. The values for each question range from 0 to 4, and final scores are anchored on a scale of 0 to 148, where 148 represents the highest quality of life. There are 7 items for physical wellbeing (PWB), 7 items for social/family wellbeing (SWB), 6 items for emotional wellbeing (EWB), 7 items for functional wellbeing (FWB), and 10 items for additional concerns about breast cancer (BCS). The total scores for each dimension are as follows: PWB, 28; SWB, 28; EWB, 24; FWB, 28; and BCS, 40. The validity and reliability of the FACT-B in the Chinese version were confirmed [25].

### Clinical data

From electronic patient records, this study obtained clinical data, including morbidity status, clinical stages, clinical practice, and menopausal status.

### Demographic determinants

Self-reported demographic determinants include patient age, education status, marriage, type of medical insurance, profession, and household income.

### Dependent variable from EQ-5D-5 L

This study used the widely validated EQ-5D-5 L to measure health utility as a dependent variable [26]. The EQ-5D-5 L comprises five self-reported dimensions and the EQ-VAS. The self-reported dimensions are mobility, self-care, usual activities, pain/discomfort, and anxiety/depression; each of these is measured along 5 levels of severity. To investigate the correlation between the FACT-B and the EQ-5D-5 L, this study assigned the values of severity to range from 5 to 1, where 5 indicates that patients can perform activities in the dimension without difficulty. Additionally, the respondents agreed to complete the EQ-VAS test to measure their health status. Values of health status are anchored on a scale of 0 to 100, where 100 represents the best health status imaginable.

### Data analysis

The value set of China was used to transfer overall scores from the EQ-5D-5 L to health utility [27].

The Wilcoxon test and the Kruskal-Wallis H test were performed to screen potential demographic and clinical determinants of health utility. Only those of statistical significance were introduced into the modelling process. The skewness of the results from the EQ-5D-5 L and the FACT-B was assessed.

The Spearman correlation coefficient was used to evaluate the correlation between the FACT-B and the EQ-5D-5 L.

Three modelling algorithms, namely, the OLS model, the Tobit model, and the two-part model, were used to develop mapping functions from the FACT-B to the EQ-5D-5 L.

OLS is commonly used in econometrics to estimate parameters by minimizing the sum of squared errors of data. Although it performs well in many fields of research, its predictability could be restricted by the scale of health utility, ranging from 0 to 1. The ceiling effects of health utility could also lead to skewed distribution and heteroscedasticity, which invalidates the normality assumption of OLS. Therefore, OLS is theoretically not the most appropriate model in mapping health utility [28]. However, OLS was concluded to be the best model in a prior study and was referred to by approximately 80% of publications conducting mapping functions with regard to health utility [17, 29, 30].

The Tobit model is an alternative that improves the ability to cope with ceiling effects. Another alternative is the censored least absolute deviations (CLAD), a median-based method. However, most econometric models are based on the mean, which is a consideration that led this study not to include or evaluate CLAD [31].

The two-part model has been suggested for use in mapping health utility due to its predictability and ability to cope with ceiling effects [32, 33]. This model is divided into two steps. The first step involves estimating the entire estimated sample to predict the occurrence of ceiling effects, usually using a logistic regression model. The second step estimates the utility value in the non-complete health state and ultimately calculates the overall utility value [34]. Separate models were conducted for each domain and for the overall scores of the FACT-B, as suggested in the literature [31, 33]. Squared terms and interaction terms of statistical significance were also examined. Three modelling approaches were performed in each model. A two-tailed *P* value of less than 0.10 was considered statistically significant.

- Model 1:Overall score of the FACT-B
- Model 2: All domain scores on the FACT-B
- Model 3: Domain scores on the FACT-B of statistical significance in model2
- Model 4: Model3 + squared terms of statistical significance in model2
- Model 5: Model 4 + interaction terms of statistical significance in model2

To compare the models, we considered their goodness of fit, applicability, and simplicity. Goodness of fit indicates the extent to which the model interprets the observed data. Mean absolute error (MAE), root mean square error (RMSE), mean absolute deviation (MAD), Akaike information criteria (AIC) and Bayes information criteria (BIC) were used as important indicators for model selection:lower MAE, RMSE, MAD, AIC and BIC represent better models. R^2^ was computed to measure the predictability of the OLS model. In the Tobit and TPM regression methods, the determination coefficient R^2^ is not clearly defined. Referring to the study by Cheung YB et al. [17] and comparing the results of the two studies, we calculated the square of the correlation coefficient (*r*) between the observed and predicted values of each model. Here, *r*^*2*^ is equivalent to *R*^*2*^ in OLS. To avoid overestimating r^2^ due to an increase in independent variables, we define the adjusted r^2^ as follows: 1- $\frac{\left(n-1\right)}{\left(n-p-1\right)}\left(1-{\mathrm{r}}^2\right)$. In this formula, *n* represents the sample size, and *p* is the number of parameters in the model. Finally, if the model shows similar MAE, RMSE, MAD, AIC, BIC and r^2^ values, applicability and model simplicity will be considered. Due to the lack of available external data in this study, 5-fold cross-validation was used to examine the stability and reliability of the model, and the result of the cross-validation was measured using the MAE.

Observed and predicted EQ-5D values were plotted to measure model performance.

We also performed non-parametric tests (Mann-Whitney U tests for two categories or Kruskal-Wallistests for more than two categories) to examine differences in EQ-5D-5 L index scores from different models by demographic and clinical features. Simple linear equating was used to model OLS 5 to avoid regression to the mean. We used the following linking function that transforms the X-scores to have the same mean and standard deviation as the Y-scores [35]:Y= ${\upmu}_{\mathrm{Y}}+\left(\frac{\upsigma_{\mathrm{Y}}}{\upsigma_{\mathrm{X}}}\right)\left(\mathrm{X}-{\upmu}_{\mathrm{X}}\right)$, where μ_X_ and μ_Y_ are the mean values of X and Y, and σ_X_ and σ_Y_ the standard deviations. The mean of the model OLS 5 was 0.857, and the variance was 0.161. Therefore , μ_X_ was 0.857 and σ_X_ was 0.161.

Data analyses were performed in Stata version 12.0(StatCorp, College Station, TX).

## Results

The average age of the 446 patients was 52.03 years (SD, 8.79). Demographic and clinical characteristics are summarized in Table 1.

**Table 1:** Demographic and clinical characteristics of the study participants

Characteristic	Groups	N	%
Age, year	<45	74	16.59
	45~54	215	48.21
	55~64	138	30.94
	≥65	19	4.26
Nationality	Han nationality	438	98.21
	Minority	8	1.79
Education level	Elementary and below	144	32.29
	Junior high school	150	33.63
	Senior high school	88	19.73
	Undergraduate or over	64	14.35
Marital status	Single	7	1.57
	Married	411	92.15
	Divorced/separated	14	3.14
	Widowed	14	3.14
Account location	Rural	220	49.33
	Urban	226	50.67
Health-care insurance	Urban employees	193	43.27
	Urban residents	54	12.11
	New rural cooperative scheme	177	29.69
	Other	22	4.93
Occupation	Public sector employee	28	6.28
	Enterprise or company employee/worker	28	6.28
	Self-employed	23	5.16
	Farmer/worker	123	27.58
	Unemployed	148	33.18
	Retiree	96	21.52
Household income in 2017, Chinese Yuan	<30,000	234	52.47
	30,000~80,000	148	33.18
	80,000~ 150,000	45	10.09
	≥150,000	19	4.26
Course of disease,month	≤12	133	29.82
	13~36	147	32.96
	37~60	78	17.49
	≥61	88	19.73
TNM stage	0	17	3.81
	I	72	16.14
	II	224	50.22
	III	99	22.20
	IV	34	7.62
Hormone receptor (ER/PR)	Positive	306	68.61
	Negative	78	17.49
	Mixed	56	12.56
	Unkonwn/missing	6	1.35
HER2	Positive	355	79.60
	Negative	81	18.16
	Unkonwn/missing	10	2.24
Inpatient/Outpatient	Outpatient	376	84.30
	Inpatient	70	15.71
Surgical therapy	Breast conserving surgery	101	22.65
	Modified radical surgery	331	74.22
	Unsurgical	14	3.14
Chemotherapy	No	37	8.30
	Yes	409	91.70
Radiotherapy	No	175	39.24
	Yes	271	60.76
Targeted therapy	No	403	90.36
	Yes	43	9.16
Endocrine therapy	No	139	31.17
	Yes	307	68.83
Menopause	No	63	14.13
	Yes	383	85.87
Disease state	Primary breast cancer within one year (state P)	125	28.03
	Primary and recurrent breast cancer for the second year and above (state S)	258	57.85
	Recurrent breast cancer within one year (state R)	20	4.48
	Metastatic cancer (state M)	43	9.64

**Fig. 1:**
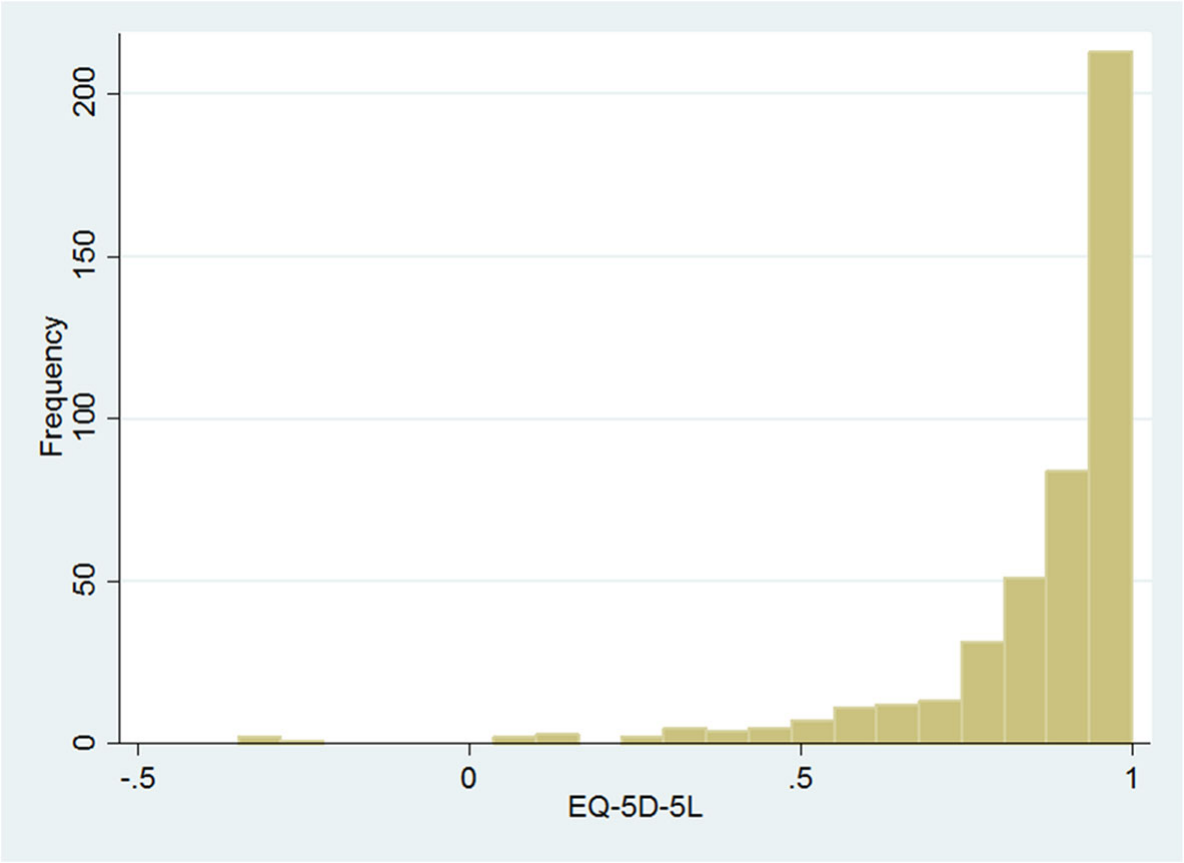
Histogram of EQ-5D-5 L scores

**Fig. 2:**
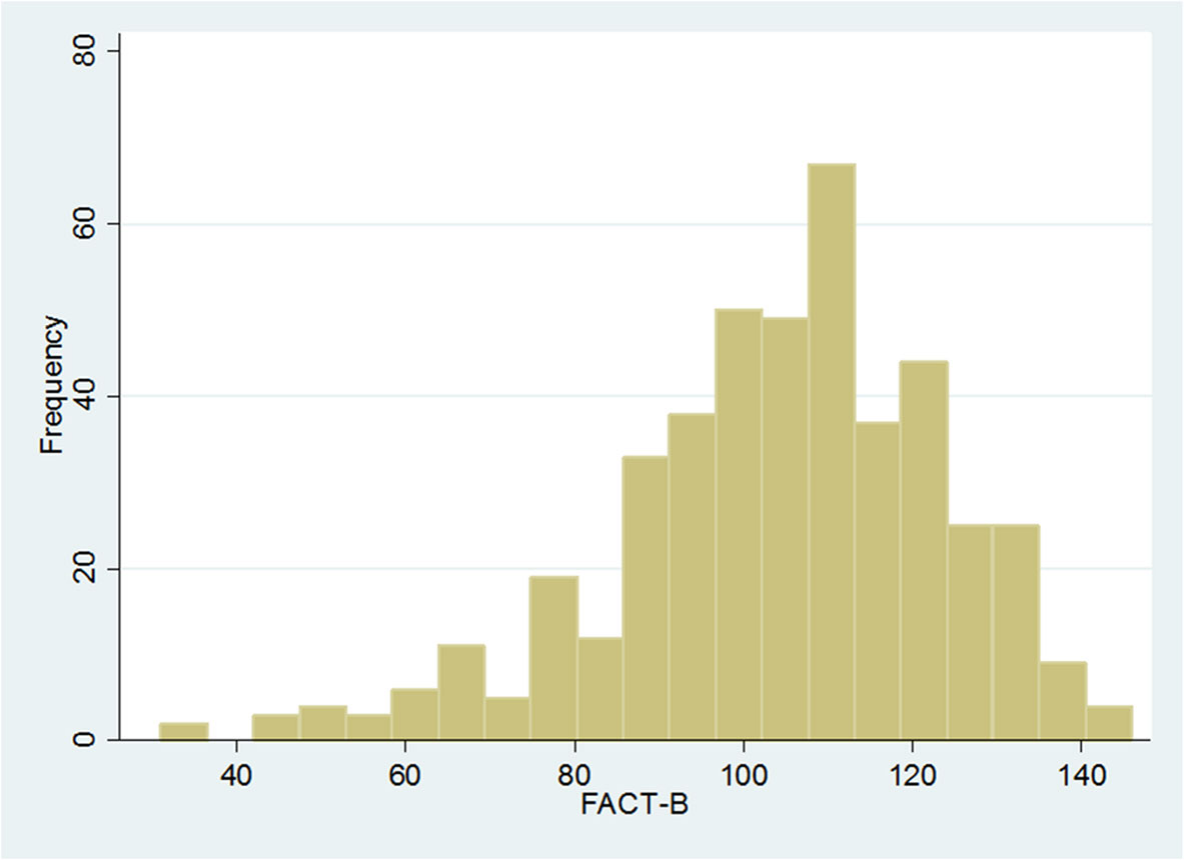
Histogram of FACT-B scores

Figures 1 and 2 present the histograms of the results from the EQ-5D-5 L and the FACT-B, both of which were skewed to the right. The values of the two measures are summarized in Table 2. The value of the EQ-5D-5 L ranged from − 0.349 to 1;the mean was 0.857(SD, 0.193), and the median was 0.197. The ceiling effects existed in the health utility of 25.11% of participants, which did not exist in the results from the FACT-B. The mean of the results from the FACT-B was 104.31(SD, 19.732), and the median was 106.

**Table 2:** Description of EQ-5D-5 L and FACT-B scale scores

Item	Mean	SD	Median	Maximum	Minimum	Flooring (%)	Ceiling (%)
EQ-5D	0.857	0.193	0.902	1	− 0.349	0	25.11
FACT-B	104.031	19.732	106	146	31	0	0

Table 3 presents the Spearman correlation coefficients between the results from the EQ-5D-5 L and those from the FACT-B. The correlation coefficient between the overall score of the two measures was 0.642. The correlation coefficients between the overall value from the EQ-5D-5 L and the values of each dimension of the FACT-B ranged from 0.389 to 0.601. The correlation coefficients between the overall score of the FACT-B and the values of each dimension in the EQ-5D-5 L ranged from 0.316 to 0.627. The correlation coefficients among each dimension from the two measures ranged from 0.203 to 0.535. The *P* value of each correlation coefficient was less than 0.001.

**Table 3:** Correlation between EQ-5D-5 L scale and FACT-B scale scores

Dimension	PWB	SWB	EWB	FWB	BCS	FACT-B total score
Mobility	0.463^*^	0.264^*^	0.316^*^	0.386^*^	0.332^*^	0.424^*^
Self-care	0.535^*^	0.203^*^	0.245^*^	0.254^*^	0.285^*^	0.316^*^
Usual activities	0.432^*^	0.334^*^	0.360^*^	0.405^*^	0.418^*^	0.483^*^
Pain/discomfort	0.469^*^	0.272^*^	0.416^*^	0.305^*^	0.425^*^	0.465^*^
Anxiety/depression	0.488^*^	0.378^*^	0.643^*^	0.428^*^	0.533^*^	0.627^*^
EQ-5D-5 Ltotal score	0.601^*^	0.389^*^	0.558^*^	0.471^*^	0.545^*^	0.642^*^

^*^P<0.001

Correlations between demographic and clinical characteristics and results from the EQ-5D-5 L were examined. Correlations between results from the EQ-5D-5 L and type of health insurance, clinical stage, TNM stage, admission, the practice of endocrine therapy, and morbidity status were statistically significant (*P* value less than 0.05). Age, minority status, education, marital status, registered location (Hukou), profession, household income, hormone receptor, HER2, surgical procedures, chemotherapy, radiotherapy, targeted therapy, and menopausal status were not found to be statistically significant.

Five models developed by OLS are presented in Tables 4 and 6. With respect to goodness of fit, models with squared terms and interaction terms performed better. Models developed by Tobit analysis and TPM are presented in Table 5. Compared with models 1 to 4, model 5 performed better for both Tobit models and TPMs. Although interaction terms and squared terms were introduced to OLS 5, Tobit 5 and TPM 5, the interaction terms of statistical significance were different in each model. PWBxBCS was statistically significant in OLS 5 and Tobit 5, while PWBxBCS and PWBxFWB were statistically significant in TPM 5.

**Table 4:** Coefficient estimates of ordinary least-square regression

Variable	OLS1	OLS2	OLS3	OLS4	OLS5
Constant	0.13583***	0.02434	0.03209	−0.73772***	− 0.9110***
FACT-B total score	0.00693***				
PWB		0.02103***	0.02101***	0.05172***	0.05918***
SWB		0.00118			
EWB		0.00368*	0.00400**	0.02837***	0.01932*
FWB		0.00386***	0.00433***	0.01108**	0.02086***
BCS		0.00722***	0.00728***	0.03138***	0.03505***
Dimension squared					
PWB squared				−0.00088***	− 0.00030
EWB squared				−0.00076***	− 0.00029
FWB squared				−0.00017***	0.00006
BCS squared				−0.00045***	0.00011
Dimension interaction					
PWB × EWB					0.00006
PWB × FWB					−0.00019
PWB × BCS					−0.00111***
EWB × FWB					−0.00019
EWB × BCS					−0.00019
FWB × BCS					−0.00036

* *P*<0.10, ** *P*<0.05, *** *P*<0.01

**Table 5:** Coefficient estimates of Tobit and Two-part model using main effects with or without interaction terms

Variable	Tobit4	Tobit5	Two-part 4		Two-part 5	
			First-part	Second-part	First-part	Second-part
Constant	−0.60177***	− 0.77881***	0.00048	− 0.81605***	4.89e-06	−1.02358***
FACT-B total score						
PWB	0.04438***	0.05329***	0.62518**	0.05972***	0.76743	0.06598***
EWB	0.02293**	0.01407				
FWB	0.00821	0.01547	0.90802	0.02163***	0.89512	0.03590***
BCS	0.02609***	0.03107***	1.55562	0.04312***	1.78533	0.04396***
Dimension squared						
PWB squared	−0.00062***	− 0.00007	1.01569***	− 0.00106***	1.01664***	−0.00033
EWB squared	−0.00057*	− 0.00023				
FWB squared	−0.00003	0.00013	1.00524	−0.00048***	1.00599*	−0.00019
BCS squared	−0.00032*	0.00018	0.99500	−0.00069***	0.99905	−0.00015
Dimension interaction						
PWB × EWB		0.00001				
PWB × FWB		−0.00013			1.00542	−0.00059*
PWB × BCS		−0.00114***			0.98847	−0.00105***
EWB × FWB		−0.00010				
EWB × BCS		−0.00005				
FWB × BCS		−0.00032			0.99508	−0.00042

* *P*<0.10, ** *P*<0.05, *** *P*<0.01

Fifteen models using three modelling approaches are presented in Table 6. Table 6 shows all 15 models of the three technology modelling constructions, namely, the OLS model, the Tobit model, and the TPM.

**Table 6:** Summary of model performance for OLS, Tobit and TPM models

Model	Model 1	Model 2	Model 3	Model 4	Model 5
OLS					
r^2^	0.503	0.602	0.601	0.676	0.700
Adjusted r^2^	0.502	0.597	0.599	0.671	0.690
RMSE	0.136	0.122	0.122	0.111	0.108
MAD	0.106	0.112	0.112	0.103	0.101
MAE	0.094	0.086	0.086	0.074	0.071
AIC	− 511.486	−602.471	− 603.658	− 687.881	−705.106
BIC	−503.286	−577.869	−583.157	− 650.979	−643.601
Tobit model					
r^2^	0.575	0.648	0.647	0.680	0.694
Adjusted r^2^	0.574	0.644	0.645	0.676	0.687
RMSE	0.127	0.115	0.115	0.109	0.107
MAD	0.099	0.106	0.106	0.103	0.102
MAE	0.084	0.078	0.078	0.073	0.072
AIC	−131.380	− 196.891	−197.837	−216.597	− 220.060
BIC	−119.079	− 168.189	− 173.235	− 175.594	−154.455
Two-part model					
r^2^	0.534	0.615	0.614	0.674	0.695
Adjusted r^2^	0.533	0.611	0.611	0.669	0.689
RMSE	0.132	0.120	0.112	0.110	0.106
MAD	0.110	0.113	0.114	0.102	0.099
MAE	0.087	0.081	0.082	0.072	0.071
AIC	− 341.450	−408.173	− 410.354	− 470.352	− 492.476
BIC	− 333.877	− 385.306	− 395.109	−443.674	− 454.365

From the perspective of *r*^*2*^, the OLS model (0.503~0.700) had the largest *r*^*2*^, in comparison to that of the TPM (0.534~0.695) and the Tobit model (0.575~0.694). In models 1–4, the *r*^*2*^ values of the OLS model and the TPM were slightly smaller than that of the Tobit model. However, it was significantly improved in model 5, exceeding that of the Tobit model. Independent variables varied among models 1–5. Model 2 generally performed better than model 1 within each domain with respect to the overall score, while model 2 performed consistently with model 3, which had fewer independent variables and was more concise. The introduction of squared terms and interaction terms in models 4 and 5 improved model performance. For the three indicators of RMSE, MAD, and MAE, the value of model 5 was the smallest. Compared with RMSE, MAD, MAE of OLS 5, Tobit 5 and TPM 5, the values of the three indicators were very similar. OLS 5 had the smallest AIC and BIC values. Thus, considering the simplicity of the model, the best-performing model of the 15 models was TPM 5.

The predicted values from model 4 and model 5 are presented in Table 7. The Tobit model had a poor prediction of the mean. While predicted values from OLS were much closer to observed values, the OLS model overestimated poor health. Moreover, 0.897% of the predicted values from OLS 4 were larger than 1. In contrast to the OLS model, the TPM performed well for lower values.

**Table 7:** Descriptive summary of EQ-5D-5 L utility index derived from observed and predicted values of best fitting models

Model	Mean	SD	Minimum	P10	Median	P90	Maximum	Upper bound(%)
Observed data	0.857	0.193	−0.348	0.642	0.902	1	1	0
OLS4	0.857	0.158	−0.213	0.663	0.905	0.972	1.005	0.897
OLS5	0.857	0.161	−0.267	0.685	0.909	0.962	0.988	0
Tobit4	0.852	0.156	−0.157	0.648	0.902	0.963	0.985	0
Tobit5	0.853	0.160	−0.212	0.674	0.906	0.962	0.979	0
TPM4	0.857	0.156	−0.178	0.668	0.904	0.971	0.992	0
TPM5	0.857	0.160	−0.305	0.685	0.906	0.964	0.986	0

Table 8 used a 5-fold cross-validation method to randomly divide the samples into 5, each of which was selected as the verification set, and the remaining 4 were used as training sets. This was repeated 5 times, and the average MAE was calculated for each. The results showed that the MAEs were 0.07155~0.08509 after the six best models were verified by 5-fold cross-validation. The TPM had the smallest MAE, followed by the OLS model and the Tobit model.

**Table 8:** Out-of sample 5-fold cross-validation of best fitting models

Model name	Mean absolute error
OLS 4	0.07495
OLS 5	0.07369
Tobit 4	0.08509
Tobit 5	0.08319
TPM 4	0.07271
TPM 5	0.07155

Table 9 shows the EQ-5D-5 L values for patients with different demographic and clinical characteristics in the three best models. The estimated health utilities from model OLS 5 were closer to the measured ones than those from model Tobit 5 and TPM 5. When the linear equated scores were used for model OLS 5, the predicted values were closer to the means of the actual values.

**Table 9:** Mean actual value and predicted value between different demographic and clinical characteristics patients in 3 best models

Characteristic	Groups	Actual value	Predicted value						Equated value	
			OLS5	*P*	Tobit5	*P*	TPM5	*P*	OLS5	*P*
Health-care insurance	Urban employees	0.871	0.870	0.002	0.866	0.003	0.869	0.002	0.872	0.002
	Urban residents	0.912	0.907		0.904		0.908		0.916	
	New rural cooperative scheme	0.825	0.826		0.822		0.827		0.820	
	Other	0.857	0.869		0.863		0.867		0.871	
Course of disease, month	≤12	0.809	0.821	<0.001	0.817	0.001	0.819	<0.001	0.814	<0.001
	13~36	0.887	0.883		0.879		0.882		0.888	
	37~60	0.912	0.896		0.893		0.895		0.904	
	≥61	0.831	0.833		0.829		0.836		0.828	
TNM stage	0	0.900	0.897	0.051	0.893	0.072	0.896	0.144	0.905	0.051
	I	0.899	0.892		0.888		0.890		0.899	
	II	0.869	0.864		0.860		0.863		0.866	
	III	0.844	0.835		0.831		0.835		0.831	
	IV	0.701	0.776		0.775		0.791		0.760	
Inpatient/Outpatient	Outpatient	0.889	0.882	<0.001	0.878	<0.001	0.881	<0.001	0.887	<0.001
	Inpatient	0.685	0.723		0.720		0.726		0.696	
Endocrine therapy	No	0.810	0.820	0.001	0.817	0.003	0.821	0.002	0.813	0.001
	Yes	0.878	0.873		0.869		0.873		0.877	
Disease state	Primary breast cancer within one year (state P)	0.814	0.818	<0.001	0.814	<0.001	0.816	<0.001	0.811	<0.001
	Primary and recurrent breast cancer for the second year and above (state S)	0.904	0.893		0.888		0.892		0.900	
	Recurrent breast cancer within one year (state R)	0.779	0.776		0.773		0.782		0.760	
	Metastatic cancer (state M)	0.737	0.792		0.789		0.803		0.779	

**Fig. 3:**
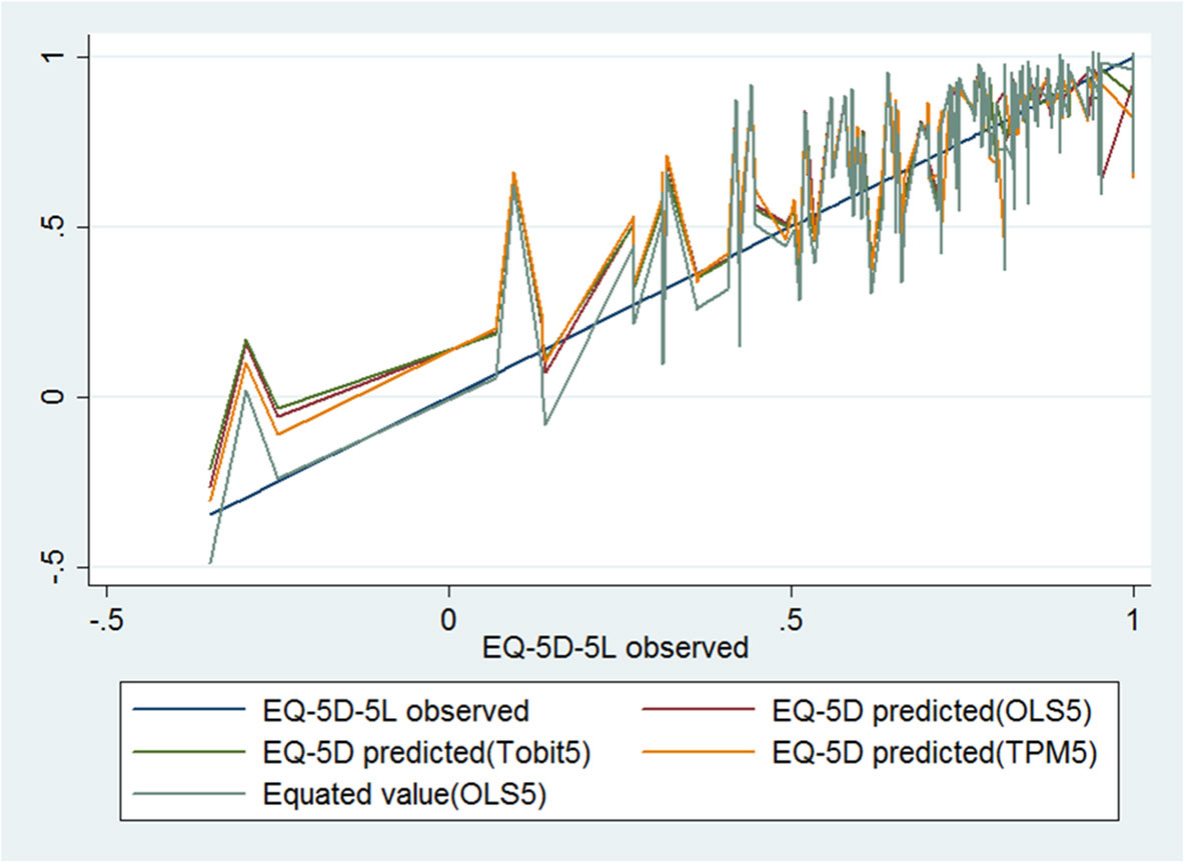
Observed and predicted EQ-5D-5 L for best fitting models for OLS, Tobit and TPM

Predicted values and observed values were plotted based on the best model among the three modelling approaches in Fig. 3. Compared with Tobit 5, the predicted values from OLS 5 and TPM 5 were much closer to the observed values from the EQ-5D-5 L. When linear equivalence was used, the value of the model OLS 5 was closest to that of the EQ-5D-5 L.

## Discussion

This study performed several mapping functions, mapping from values of the FACT-B to the health utility of the EQ-5D-5 L. Three modelling approaches, namely, OLS, Tobit, and TPM, performed heterogeneously with respect to r^2^. From the perspective of r^2^ and adjusted r^2^, the OLS model is the largest in model 5. Three modelling approaches performed similarly in the RMSE, MAD and MAE in model 5. The AIC and BIC of model OLS 5 were the smallest, although the TPM achieved a slightly smaller MAE value than the OLS model at the 5-fold cross-validation. Considering the comprehensive performance and simplicity of the OLS model, OLS 5 was selected as the best model (r^2^ = 0.700, adjusted r^2^ = 0.690, RMSE = 0.108, MAD = 0.101, MAE = 0.071, AIC = -705.106, BIC = -643.601).

To the best of our knowledge, this is a pioneering study for conducting mapping functions based on data from breast cancer patients in China. Although extensive research has been conducted onhealth utility mapping functions, only three prior studies performed mapping functions for breast cancer patients [16]. One study used adjusted limited dependent variable mixture models (ALDVMMs) to establish mapping between the FACT-B and the EQ-5D-3 L scales [19]. However, we chose a 5-dimensional scale to avoid ceiling effects. The only two articles that used the EQ-5D-5 L scale were from the same study and were based on data from 238 Singaporean women [17, 18]. Five regression models mapping from the FACT-B to the EQ-5D-5 L were conducted, which may or may not set the upper limit of health utility to 1 [17]. The OLS model, which had the best performance in the Singaporean study, performed better in this study with respect to goodness of fit(r^2^ = 0.497, adjusted r^2^ = 0.489, RMSE = 0.013, MAD = 0.091). Consistent with prior studies, the Tobit model presented lower predictability in our study [17].

Although the EQ-5D-5 L mitigated ceiling effects and floor effects, compared with the EQ-5D-3 L, the ceiling effects still existed for 25.11% of participants in the current study. The skewed distribution of EQ-5D-5 L values is presented in Fig. 1. The TPM was conducted separately for health utility values of 1 and other values to cope with data limitations, which was consistent with prior studies [36]. In Table 7, we also found that the TPM has a better predictive effect than the OLS model on larger values. However, Table 9 shows that the OLS regression had the most accurate means by different demographic and clinical characteristics, and the linear equated scores were more similar to the observed scores.

Correlation coefficients among domains of the EQ-5D-5 L and the FACT-B were assessed in this study, and the correlation coefficients were statistically significant (*P* values less than 0.001). In the past, there were mapping studies to explore the correlation between scales [37, 38]; in the case of a conceptual overlap between the two tools, the mapping is more likely to succeed. It is notable that the values of SWB did not predict health utility from the EQ-5D-5 L in the OLS model, which may result from the lack of domains related to social function onthe EQ-5D-5 L. Similarly, SWB was not statistically significant in prior mapping studies of lung cancer, prostate cancer and breast cancer [17, 39, 40].

A systematic review reported that R^2^ for the mapping function from a specific questionnaire to a generic health utility measure usually ranged from 0.4 to 0.6 [28]. The introduction of squared terms and interaction terms could significantly increase R^2^ to 0.8, which suggested that the association was non-linear [41, 42]. The introduction of squared terms and interaction terms in models 4 and 5 in our study improved model performance. The r^2^ of the model OLS 5 reached 0.700, which indicated good results. In addition, in contrast to prior studies [17], this study selected its value set in China instead of using a non-native crosswalk project to convert the EQ-5D-3 L and the EQ-5D-5 L, thus leading to improved model performance.

Overall, the mapping functions performed well in this study. Although the predicted average health utility of the OLS models much closer to the observed values, OLS would overestimate poor health and underestimate higher health utility, which was consistent with prior studies [33, 43, 44]. To solve this problem, we used simple linear equating to avoid regression to the mean [35]. This method achieved similar results in previous mapping studies [18, 45]. As shown in Fig. 3, model OLS 5 had the closest predictive values to actual EQ-5D-5 L scores after a linear equivalent method was applied.

The present study suffers from several limitations. First, the study was based on a small sample size from a single hospital. Future studies should consider collecting data from larger sample sizes and from multiple treatment centres. Second, it is impossible to conduct cross-validation for external validity in independent data sets. Therefore, although cross-validation is considered to be “second best”, it was used in this study due to a lack of external data sources. Finally, the study only used three modelling approaches in mapping functions; many advanced technologies, including the three-part model and the probit mapping function based on Bayesian networks, could be potential alternative approaches to mapping functions.

## Conclusion

Mapping is primarily used to obtain utility scores from disease-specific non-preference tools, allowing a large amount of existing survey data to be used for economic analysis. The use of mapping algorithms has facilitated the development of cost-utility research. To the best of the author’s knowledge, this study is the first to develop a mapping algorithm between the FACT-B and the EQ-5D-5 L in the Chinese patient population and to adopt the recently developed EQ-5D-5 L tariff value based on Chinese population preferences. The best model for estimating the EQ-5D-5 L value includes the FACT-B subscale scores. The addition of squared terms and interaction terms improves the predictability of the model. When using several algorithms developed by these data, we found that the prediction performance of the OLS model was better than that of the Tobit model and the TPM. It is hoped that this algorithm will help to develop cost-utility studies to evaluate breast cancer treatments in China’s healthcare environment.

## Data Availability

The dataset are available from the corresponding author on reasonable request.

## Abbreviations

AIC: Akaike information criteria
BCS: Additional concerns for breast cancer
BIC: Bayes information criteria
CLAD: Censored least absolute deviations
EQ-5D: EuroQol-5 dimension
EWB: Emotional wellbeing
FACT-B: Functional Assessment of Cancer Therapy – Breast
FWB: Functional wellbeing
MAD: Mean absolute deviation
MAE: Mean absolute error
OLS: Ordinary least-square
PWB: Physical wellbeing
QALYs: Quality-adjusted life years
RMSE: Root mean squared error
SWB: Social/family wellbeing
TPM: Two-part model
